# Defining benchmarking in the context of safety assessment of personal care and cosmetic products using New Approach Methodologies

**DOI:** 10.1016/j.namjnl.2026.100111

**Published:** 2026-07-07

**Authors:** Huang Huang, Gertrude-Emilia Costin

**Affiliations:** Institute for In Vitro Sciences, Inc. (IIVS), 30 W Watkins Mill Road, Suite 100, Gaithersburg, MD, 20878, USA

**Keywords:** Benchmarks, Cosmetics, Safety assessment, New approach methodologies (NAMs)

## Abstract

The concept of benchmark materials in the personal care and cosmetic industry is often based on the positioning of prototypes relative to the leading brand within a given product category. The main points of comparison are the market ranking, performance regarding consumers’ perception, aesthetics, cost, complexity of the manufacturing process and patents covering the product and/or its components. Even though benchmark materials are often included when conducting safety tests of cosmetic and personal care products, the concept is rather loosely defined in toxicological profiling. The use of benchmarks becomes of particular interest when safety testing is based on New Approach Methodologies (NAMs) (*in silico, in vitro* or *ex vivo* technologies) that allow for toxicity ranking of the prototypes and provide mechanistic insights without having an established prediction model. It is therefore important to define the properties and performance of the benchmark materials to better guide the safety testing of candidate formulations. In this review manuscript we discuss the criteria to select benchmarks, and provide specific examples of such finished products incorporated in the safety assessment studies of a wide range of cosmetic and personal care product classes. We also discuss strategies using benchmarks to evaluate the predictive capacity of test systems and methodologies that do not rely on the use of animals for safety assessments.

## 1 Introduction

The current cosmetic and personal care industry market is more expanded than it has ever been in terms of the wide range of products launched at fast pace and the innovations behind the mechanisms of action of the ingredients used. The modern day consumer relies on the duty of manufacturers to execute their due diligence regarding the safety and efficacy assessment of these products before making them available to the masses for use.

Within this context, the prohibition of animal testing for cosmetics by the European Union represents one of the most significant regulatory and ethical transformations in consumer product safety assessment (European Union, 2023). The implemented legislation does not allow the testing of cosmetic ingredients and finished products on animals, nor the marketing of products developed using animal testing. This significant regulatory change fundamentally reshaped cosmetic safety assessment by accelerating the development, validation, and regulatory acceptance of NAMs. As cosmetic products become more complex and regulatory expectations continue to evolve, the successful implementation of NAMs depends on rigorous quality systems, appropriate benchmark materials, and transparent demonstration of their applicability domain, reproducibility, and biological relevance. Consequently, NAMs are not only replacing animal tests but are also enabling more mechanistically informed, human-centered safety evaluations that align with both scientific advancement and ethical principles.

The continued expansion of animal testing bans worldwide is expected to further stimulate innovation in predictive toxicology and promote the widespread adoption of human-based testing strategies that improve both scientific relevance and consumer safety.

As cosmetic and personal care industries are differently regulated compared to others (food, pharmaceutical, agrochemicals, etc.), the selection of laboratory methodologies and tests to assess safety endpoints becomes the responsibility of the toxicologists working for the manufacturer and/or for the Contract Research Organizations (CROs) conducting the testing. Often times, during the design of the safety assessment studies the toxicologists refer to established products on the market as point of reference or comparison. Of interest are especially those very well characterized from a safety perspective and that are qualified as reliable for relevant paired data analysis. However, there is a lack of understanding of what a reference or benchmark material is, what is its role and how it can be used to evaluate the toxicological profile of prototypes. In our efforts to identify the industry lexicon of terms, we came across an array of definitions which we will analyze in the manuscript as they are used relative to pharmaceutical, botanical or cosmetic products. We also provide examples of established benchmarks for various product lines (raw ingredients, home cleaning, personal care, intimate care and oral care products, respectively) and safety assessment endpoints such as ocular, dermal, mucosal irritation, skin sensitization or phototoxicity.

Our goal is to provide clarity in regards to the definition of benchmarks captured based on the understanding of this concept within different industry sectors. We also expand on their profile, criteria for selection and inclusion in safety testing based on NAMs, and on the utility of benchmarks beyond the conduct of targeted toxicological assessments.

## 2 Benchmarks definitions

The concept of benchmarks may have loosely evolved from the reference standards, with an implied understanding that benchmarks may often be complex formulations or finished products. Generally, a reference standard is defined as a substance which has a known level of toxicity determined *in vivo*, and which can be used *in vitro* to evaluate the toxicity levels of prototypes, whose effects are scaled relative to the reference standard (Balls and Fentem, 1999). In industry, reference standards are already widely used for making safety decisions regarding the acceptability of new formulations and for prioritizing product development and innovation. For example, if *in vitro* skin irritation data exist for two reference standards designed and demonstrated to cover both ends of the toxicological profile (irritant and non-irritant, respectively), it should be possible to classify a prototype by comparing its results generated *in vitro* with the *in vitro* results of the two reference standards. It should also be possible to obtain a measure of confidence for the classification according to the proximity of the *in vitro* result to each boundary, and especially when established prediction models exist.

From a regulatory perspective, although benchmarks are not required by Good Laboratory Practices (GLPs), industry has found it beneficial to add them concurrently to studies submitted to regulatory agencies. For example, the inclusion of a product registered as an U.S. Environmental Protection Agency (EPA) Category II eye irritant as a benchmark material in an *in vitro* eye irritation study will establish the response from the *in vitro* test system against which a prototype can be evaluated. If the prototype is predicted to be a Category II eye irritant, and its results are similar to those of the benchmark, then there can be a high level of confidence that the test system is responding appropriately to the chemical class of interest, and that the irritation prediction is reliable. However, industry concerns exist when including for testing marketed products as benchmarks, and especially in the categories regulated by the Federal Insecticide, Fungicide, and Rodenticide Act (FIFRA). Specifically, the 6(a)2 FIFRA provision requires the registrants to submit factual information to the U.S. EPA about new information acquired and regarding unreasonable adverse effects on the environment induced by their products. This provision may therefore affect the existing products if new data are generated to indicate adverse effects not previously identified (U.S. EPA, 1998). This however is not interpreted as an equally risky approach by the personal care or cosmetic industry which are differently regulated.

We share in the following sections the definitions and uses of benchmark materials across several industry sectors and in relation to pharmaceuticals, botanicals, personal care and cosmetic products.

### 2.1 Benchmarks in pharmaceutical industry

The pharmaceutical industry has a long history of benchmarks use for safety and efficacy evaluation of their products and activates within the regulatory space for an equally long time. Even though the pharmaceutical compounds and products are not within this manuscript’s focus, we will discuss the benchmarks in that context for the opportunity to compare and contrast the understanding and use of the concept across industries.

The terminology used in the pharmaceutical industry regarding the selection of relevant products for comparative safety assessments is quite different from the cosmetic industry. The pharmaceutical industry uses what is called a “comparator pharmaceutical product (or compound)” rather than a “benchmark”. Such selection is more often made for clinical studies than for pre-clinical assessments and it may be based on the patients’ experience with a product (*e.g.*, injection) and successful impact in the clinical setting. Regarding the safety testing using NAMs, the landscape of comparators is slightly different. For example, compounds that failed the safety in pre-clinical (often *in vivo*) or clinical testing will be considered as benchmarks of toxicity for the next generation of similar compounds to support the screening of toxic compounds and avoid failures occurring later, during the clinical trials. For efficacy testing, the selection of a comparator is dependent on the stage and development of a novel compound to be assessed. The hazard testing is also based on whether an active pharmaceutical ingredient (API) or an intermediate compound synthesized at various stages in the synthesis of the API is the subject of the safety evaluation. Regarding drugs’ efficacy, so called “tool compounds” are used in the pharmaceutical industry to assess potency of novel molecules.

World Health Organization (WHO) established guidance documents on criteria to be used for the selection of comparator products (WHO, 2015). Before 1996, the criteria of a comparator product selection was either based on being the most widely used (leading) product on the market or on the product being the first one in its class introduced for use. Starting with 2006, the concept of multisource (generic) pharmaceutical products was introduced. They were defined as products to conform to the same appropriate standards of quality, efficacy and safety as those applicable to the innovator’s product. Also, reasonable assurance should be provided within this context that the multisource product is therapeutically equivalent and interchangeable with the comparator product.

In most therapeutic areas, multiple drug options are increasingly becoming available, but there is often a lack of evidence from head-to-head clinical trials to allow for a direct comparison of the efficacy (and/or safety) of one drug *vs.* another. The situation arises partly from drug registration in many worldwide markets being only reliant on demonstrated efficacy from placebo-controlled trials. Furthermore, trials with active comparators designed to show non-inferiority or equivalence of one drug *vs*. another, generally need large sample sizes, and hence are expensive to conduct. In the absence of head-to-head clinical trial data, several statistical methods have been developed over the past 15 years to allow for indirect comparison between or among various drugs (or other interventions). Four of these statistical methods are reviewed in the manuscript by Kim et al., 2014 that conducted also an analysis of pharmaceutical comparators. The naïve direct comparison between two drugs is an analysis based on clinical trial results for one drug that are directly compared with clinical trial results for another drug; in this scenario, there is no attempt to adjust for any discordance in comparators between/among the trials. This analysis is in most instances considered inappropriate and should only be used for exploratory purposes and in the absence of any other better options. Another analysis is called adjusted indirect comparison with a common comparator. It compares the magnitude of the treatment effect between two treatments relative to a common comparator, which serves as a link between the two treatments. When no common comparator can be identified between two drugs of interest, a series may be constructed whereby the two drugs are linked indirectly *via* two or more comparators (multiple adjusted indirect comparison); in this scenario, uncertainty accumulates at every link. Finally, the mixed treatment comparison is based on the concept that any comparison that includes either one of two drugs being compared contains information that can be used to describe the link between the pair. Of all these methods, the adjusted indirect comparison is currently the most commonly accepted method. To reduce uncertainty, the mixed treatment comparison method uses Bayesian statistical models to incorporate all available data for a drug. However, this method has not yet been widely accepted by researchers, nor by drug regulatory and reimbursement authorities.

### 2.2 Benchmarks in relation to botanicals

Within the realm of botanicals that are often used in the composition of cosmetic and personal care products, several manuscripts explored the concept of comparators (Schilter et al., 2003; Antignac et al., 2011; Neely et al., 2011; Yimam et al., 2018; Wang et al., 2022). In general, there is agreement regarding the strategy to be used for the safety assessment of botanicals based on all existing knowledge on adequate comparators with a history of human use. Comparison requires detailed background and compositional information on the newer product and comparators, an assessment of the history of use of the traditional products and a description of the intended use and consequent exposure of the newer products. Such an approach is considered as a good starting point to identify potential hazards and to determine the need for further information such as experimental toxicological data. A comparative approach may be completed by *in silico* analyses, such as chemical grouping and read-across approaches. For substances used in small concentrations that are predicted to produce minimal systemic human exposure and induce no local adverse effects or intolerance, the concept of the Threshold of Toxicological Concern (TTC) may be applied. The usefulness of this approach has been recently demonstrated for the systemic safety assessment of Calendula extracts (Re et al., 2009). The Dermal Sensitisation Threshold (DST) may be another pragmatic tool that can be used to prioritize the performance of local toxicity studies (Safford, 2008). Gao et al., 2024 proposed a complex Weight of Evidence (WoE) approach that integrated human clinical and animal data alongside results generated in validated [Direct Peptide Reactivity Assay (DPRA), human Cell Line Activation Test (h-CLAT) and KeratinoSens™) and non-validated NAMs (Sens-IS). In their experiments, the authors established a group of 14 unique botanicals that can be considered a reference set based on their data rich profiles in terms of sensitization potential and potency. This set can therefore be used to evaluate existing or to develop new methods for this endpoint.

### 2.3 Benchmarks in personal and cosmetic industries

The primary understanding of a benchmark material in the personal care and cosmetic industry is based on the marketing approach that would select such a product as the target to be outperformed by prototypes. The evaluation criteria include the market position, performance, aesthetics, cost, manufacturing process and existing patents (Romanowski, 2013). This view greatly restricts the horizon regarding the safety or efficacy evaluation of prototypes, providing little to no guidance on how to select a relevant benchmark for pre-clinical testing. Of the enumerated characteristics, performance comes close to providing insight into the selection process as identified for the series of lab and/or consumer tests intended to evaluate which benchmark is the leading option.

Discussions with experienced representatives of cosmetic and personal care industry provided more context into the selection and qualification process of benchmarks for testing purposes. For example, considerations in the selection of a benchmark are often based on whether an individual chemical or a finished product is tested as prototype. Other criteria take into account whether the prototype is a modification of an existing marketed product or a new(er) product category. Some manufacturers often use their own marketed product (previously or currently on the market, similar in composition and exposure to the prototype being assessed). Ideally, substantial safety information (internally generated or from published literature) and in market surveillance data should exist for this type of benchmark. In some cases, a competitor’s marketed product may be selected as an acceptable benchmark if it has an apparent history of safe use in the marketplace. In this case, there is limited to no access to the complete composition, level of ingredients or available safety data on the product in the public domain. An assumption is made in these instances that products which reached the market have been thoroughly evaluated by their manufacturers for their claimed safety and efficacy. Occasionally, prototypes previously determined to be borderline or not acceptable in terms of safety may be used for testing as benchmarks not meeting that criteria to help define the acceptable range for the new material. In this scenario, it is advisable to pair the testing with an acceptable benchmark to help narrow down where on the safety spectrum the new prototype would be situated. If the test material is a chemical (*e.g.*, surfactant), a structurally similar analog or one from the same chemical or functional class may be used, especially if safety data exist for its toxicological profile. Last but not least, it is not uncommon to include more than one benchmark in a study, dependent on existing safety data on the test material or purpose for the study (objective safety, regulatory requirements, etc.).

## 3 Examples of benchmarks types and uses

Through the review of available sources, 32 unique ones were retrieved from the public domain (the vast majority peer-reviewed manuscript and posters), and were identified to report on the inclusion of benchmarks for the safety evaluation of prototypes. Of the 32 sources, 6 reported on products referenced in more than one category (Doucet et al., 2006; Walters et al., 2016; Kose et al., 2018; Ma et al., 2021; Roso et al., 2021; Reynolds et al., 2022). As summarized in Table 1, 8 sources were analyzed for raw ingredients (Supplementary Table 1), 5 for household cleaning products (Supplementary Table 2), 11 for cosmetic and personal care products (Supplementary Table 3), 5 for intimate care products (Supplementary Table 4), and 6 for oral care products (Supplementary Table 5), respectively. The search and identification of these manuscripts was conducted using primarily the PubMed database and the individual websites of the journals the papers were published in or the websites posting presented posters. The search terms used to narrow the resources were: “benchmark (material)”, “reference (material)”, “raw ingredient”, “cleaning products”, “cosmetics”, “personal care products”, “shampoo”, “lotion”, “personal lubricants”, “mouth rinse” “toothpaste”, “*in vitro*”, “NAMs”, “*in vivo*”, “clinical”, “*ex vivo*”, “skin sensitization”, “sensitizers”, “regulatory”, “validated”, “non-validated”, “three dimensional (3D) tissue models”, “safety”, “ocular”, “dermal”, “oral”, “vaginal”, “Bovine Corneal Opacity and Permeability (BCOP)”, “Skin Irritation Test (SIT), “viability”, “3-(4,5-dimethylthiazol-2-yl)-2,5-diphenyltetrazolium bromide (MTT)”, “histology”, “cytokine”, “Draize”, “cytotoxicity”, “Human Repeated Insult Patch Test (HRIPT)”, “Local Lymph Node Assay (LLNA)”.

**Table 1:** Summary of resources reporting on the use of benchmarks for safety testing.

Product class	Number of resources	Detailed in:
Raw ingredients	8	Supplementary Table 1
Home cleaning products	5	Supplementary Table 2
Personal care products (surfactant-based, hair products, skin care)	11	Supplementary Table 3
Intimate care products	5	Supplementary Table 4
Oral care products	6	Supplementary Table 5

Note: each number reflects the unique references included in the respective Supplementary Table.

As for the inclusion criteria, the manuscripts considered for the analysis had to associate the product classes (of choice, see also the exclusion criteria) and the NAMs capable to address safety assessment targeting various endpoints (organ-related). Also considered were the manuscripts reporting on both NAMs validated and not validated for regulatory purposes. The manuscripts that met the following exclusion criteria were not considered for our analysis: non peer-reviewed; investigating product classes other than cosmetics, personal care, household cleaning products, oral and intimate care and raw ingredients relevant to these types of formulations.

Even though cosmetic and personal care industries have a history of benchmarks use for safety evaluations, reports have not been gathered into a collection that can be used as a source of inspiration for similar studies that need to be conducted. In this section, we identified, grouped, and analyzed all publications that report on such uses of benchmarks for the safety evaluation of various product classes. They are presented in the supplementary tables based on the type of products the benchmarks are used to evaluate and further by endpoint of interest (ocular, dermal, mucosal irritation, etc.). In the following sections, we discuss these categories of benchmarks from the perspective of how they are used to rank order the safety of the investigated prototypes. We also detail on how they can be employed to assess NAMs’ performance and reproducibility over time.

### 3.1 Raw ingredients

Discrete, well-characterized chemicals, many of which represent raw ingredients used in finished formulations, have been widely applied in the development and evaluation of *in vitro* toxicology assays across endpoints, such as dermal and ocular irritation, skin corrosion, and sensitization. A total of eight studies were identified in the public domain (Supplementary Table 1), in which chemicals from defined classes were used to assess assay(s)’ performance. The endpoints investigated were primarily skin irritation (Jirova et al., 2007; Walters et al., 2016; Sugiyama et al., 2019; Líšková et al., 2020; Kojima et al., 2021) and phototoxicity (Jirova et al., 2007; Líšková et al., 2020). Additional studies addressed oral (Bacca and Jewell-Motz, 2015) and ocular (Stern et al., 1998; Doucet et al., 2006) safety. In these studies, *in vitro* responses were typically evaluated in comparison with available animal and/or human data to assess concordance. Most experimental designs employed reconstructed human tissue models and key endpoints included tissue viability measured by MTT, supplemented in some cases by cytokine release and histological evaluation. Within this context, raw ingredients were used primarily as reference materials. They were also selected to support evaluation of the predictive capacity of the test systems and to calibrate assay performance against existing datasets. The choice of ingredients was generally aligned with their functional use in formulations (*e.g.*, surfactants, alcohols, biocides), thereby maintaining relevance to cosmetic and personal care applications.

The use of raw ingredients as benchmarks was also reported in relation to quasi drugs in Japan (Sugiyama et al., 2019). These products are regulated by requirement of data from primary skin irritation tests using rabbits based on a 24h exposure, and used as evidence for 24h closed patch tests in human. In this study, the validated Skin Irritation Test (SIT) based on the Organisation for Economic Co-operation and Development Test Guideline (OECD TG) 439 (OECD, 2025) was conducted using the following test systems: EpiSkin™, SkinEthic™ RHE, EpiDerm™ and LabCyte EPI-MODEL24. A total of 40 chemicals served as reference and included 12 surfactants, 23 oils and an array of acids, polymers and powders. The data analysis concluded that the correlation between the human patch test and the *in vitro* test methods was better than that of the primary skin irritation test using rabbits exposed for 24h. This study also showed that the methods based on reconstructed skin models were useful for evaluating human skin irritation.

### 3.2 Benchmarks used for safety assessment of home cleaning products

For the safety evaluation of the home cleaning products, 5 manuscripts were identified to have incorporated finished formulations as benchmarks in the testing (Ippolito et al., 1999; Cuellar et al., 2002; Cater and Harbell, 2013; Swanson et al., 2016; Fowler et al., 2017) (Supplementary Table 2). Given their composition, particularly the presence of surfactants, these formulations were expected to exhibit a range of irritation potentials. Ocular safety was primarily evaluated using the BCOP assay (Ippolito et al., 1999; Cuellar et al., 2002; Cater and Harbell, 2013; Swanson et al., 2016). Dermal effects were assessed using reconstructed human epidermis models (*e.g.*, EpiDerm™) (Fowler et al., 2017). *In vitro* responses were generally interpreted in the context of available *in vivo* Draize data and, in some cases, supported by historical or market data.

For these product classes, the benchmarks were used to assess the reproducibility of the test system over time (Ippolito et al., 1999), to determine the optimal exposure time to the test system or to establish a standard for an acceptable level of eye irritation potential (Cuellar et al., 2002; Cater and Harbell, 2013). In addition, such benchmarks contributed to the calibration of assay outputs by anchoring responses to products with known safety profiles, thereby facilitating interpretation of irritation potential for complex mixtures. In some instances, the benchmark products were further used to establish internal scoring systems or thresholds to guide subsequent testing.

The selection of benchmarks was typically aligned with the intended product category, enabling direct comparison between prototypes and commercially relevant formulations. Under these conditions, testing of benchmarks alongside new formulations supported comparative screening within the same product class**,** particularly in the development of milder products such as detergents designed for sensitive skin (Fowler et al., 2017). Repeated inclusion of the same benchmarks also enabled the development of longitudinal datasets, supporting trend analysis and improved confidence in assay performance.

In addition to their application in *in vitro* systems, benchmark-based approaches have been extended to clinical evaluation strategies. For example, Frederick et al., 2017 described a stepwise assessment of laundry detergent formulations in which new products were compared with marketed benchmarks having established safety profiles determined by human clinical studies. The results demonstrated comparable skin compatibility between formulations, supporting the relevance of benchmark-driven comparative approaches for evaluating product mildness and consistency across testing paradigms.

### 3.3 Benchmarks used for safety assessment of personal care products

Finished formulations and, in some cases, ingredient-informed datasets have been widely incorporated as benchmarks in the evaluation of *in vitro* assays for personal care products across multiple categories, including surfactant-based products, hair care, and skin care. For these categories, a total of 9 publications were identified in the public domain (Cater et al., 2001; Doucet et al., 2006; Vavilikolanu et al., 2007; Vavilikolanu et al., 2008; Vavilikolanu et al., 2009; Cameron et al., 2013; Walters et al., 2016; Kose et al., 2018; Ma et al., 2021; Wang et al., 2021; Reynolds et al., 2022), with 4 addressing multiple product classes (Supplementary Table 3). The primary endpoints of interest were dermal and ocular irritation, reflecting the intended application of these products and frequency of these targeted safety assessments. NAMs test systems were predominantly based on reconstructed human tissue models, with endpoints including tissue viability (MTT) and, in some cases, cytokine expression. Exceptions included the use of BCOP for specific product types (*e.g.*, liquid hand soaps) (Cater et al., 2001), as well as emerging approaches such as *in silico* platforms for skin sensitization assessment (Reynolds et al., 2022).

In these manuscripts, *in vitro* data were frequently interpreted in the context of existing datasets, including animal studies (Cater et al., 2001; Doucet et al., 2006; Ma et al., 2021; Reynolds et al., 2022), clinical human data (Cameron et al., 2013; Walters et al., 2016; Reynolds et al., 2022), and market or post-market surveillance information (Cameron et al., 2013). This integration enabled assessment of concordance between NAMs’ outputs and real-world safety profiles, supporting evaluation of the predictive performance of the test systems.

Within this framework, benchmarks were applied with multiple purposes depending on study design. They were used to support evaluation of test systems’ performance, including optimization of exposure conditions (Cater et al., 2001) and confirmation of assay responsiveness across diverse formulation types (Doucet et al., 2006; Kose et al., 2018; Ma et al., 2021; Reynolds et al., 2022). In addition, benchmarks contributed to the calibration of *in vitro* responses by anchoring results to products or ingredients with known safety profiles. This approach facilitates interpretation across a broad range of product formats (*e.g.*, gels, sprays, depilatories, baby products, and products for sensitive skin). In some cases, benchmark-informed datasets were also used to guide progression to subsequent clinical studies, linking *in vitro* findings to human-relevant outcomes (Supplementary Table 3).

Although several studies incorporated commercially available products for comparison, these were not always explicitly defined as benchmarks. This observation suggests that the systematic use of benchmarking strategies in this space remains variable (Supplementary Table 3). Nevertheless, when applied, benchmark-based approaches enabled comparative screening of new formulations within the same product category, supporting product development and refinement.

Use of benchmarks extends beyond *in vitro* systems, into clinical and translational evaluation frameworks. For example, Li et al., 2022 described an iterative safety assessment approach for baby wipes. As such, the prototypes were evaluated alongside marketed products with established safety profiles across *in vitro*, clinical, and in-use studies. Similarly, Schuler et al., 2021 demonstrated the use of reference compounds as benchmarks to confirm the suitability of reconstructed skin micronucleus assays for detecting aneugenic effects of topically applied substances. Together, these examples highlight the role of benchmarks in bridging NAM-based assessments with clinical relevance and application-specific safety evaluation.

### 3.4 Benchmarks used for safety assessment of intimate care products

A total of 5 resources reported on the use of benchmarks to assess the safety of products intended for application on the vaginal area (Supplementary Table 4). Within this group, both marketed products (Ayehunie, 2006) and reference formulations (Cunha et al., 2014) were used to support assay performance assessment. Benchmarks were selected to cover a spectrum of irritation levels but primarily addressed mild materials. Spermicides containing various concentrations of Nonoxynol-9 (N-9) were also considered as benchmarks for the other end of the spectrum, given the established irritation profile of this product class. The use of N-9 is rather unique for this category of products because it is not directly comparable with personal lubricants or vaginal moisturizers. Rather, its use got implemented in laboratory tests using NAMs based on its known irritating profile.

The NAMs used for the evaluation of intimate care products were primarily based on commercially available 3D tissue models (Ayehunie et al., 2006; Ayehunie et al., 2017; Roso et al., 2021) or explants (Dezutti et al., 2012); several experiments were conducted using cell lines (Dezzutti et al., 2012; Cunha et al., 2014). Endpoints were based on tissue or cell viability (Ayehunie et al., 2006; Dezzutti et al., 2012; Cunha et al., 2014; Ayehunie et al., 2017; Roso et al., 2021). Within this context, benchmarks were mainly used to assess the predictive capacity of the *in vitro* test systems selected for safety assessment. They also supported the rank-ordering of irritation potential induced to the test system by the prototypes investigated. For the evaluation of this product class, the concept of paired benchmarks was introduced to allow the coverage of the entire irritation spectrum (Ayehunie et al., 2006; Cunha et al., 2014).

In addition, specific benchmark formulations such as the “Universal Placebo” gel have been used to provide a consistent low-irritation reference point. Originally developed for clinical microbicide trials (Tien et al., 2005), such placebo formulations were designed to be biologically inert while maintaining physicochemical properties relevant to vaginal application. Their incorporation into *in vitro* studies enabled calibration of assay responses against a defined non-irritating baseline, facilitating comparisons across studies and platforms (Cunha et al., 2014). Using Universal Placebo as benchmark also demonstrates alignment between *in vitro* and clinical evaluation frameworks (Schartz et al., 2007). Studies by Machado et al., 2019 used marketed pharmaceutical vaginal products alongside established reference materials (*e.g.*, Universal Placebo as a negative reference and Replens® as a positive reference). The authors showed that paired *in vitro* - clinical data can be used to calibrate assay performance and support interpretation of product safety. These approaches highlight the utility of benchmarks in linking NAM outputs to clinically relevant outcomes. This is particularly important for products where some degree of *in vitro* response may be expected due to their pharmacological function.

Overall, benchmarks in intimate care product testing serve a dual role. They support evaluation and calibration of NAM-based test systems using materials with known safety profiles. Furthermore, they enable comparative screening and ranking of new formulations within a defined irritation spectrum, thereby facilitating informed decision-making in product development.

### 3.5 Benchmarks used for safety assessment of oral care products

A total of 6 resources were identified to have used benchmarks to evaluate the safety profile of oral care products. They ranged from various types of toothpastes to mouth rinse formulations (Klausner et al., 2007; Koschier et al., 2011; Wurzburger et al., 2011; Martinez et al., 2014; Roso et al., 2021; Reed et al., 2023) (Supplementary Table 5), in which both finished products (Klausner et al., 2007; Koschier et al., 2011; Martinez et al., 2014; Reed et al., 2023) and ingredients-informed formulations (Koschier et al., 2011; Wurzburger et al., 2011; Roso et al., 2021) were used to assess assay performance. These products often contained functional components such as surfactants, whitening agents, and flavoring systems, and were primarily evaluated using reconstructed oral or gingival tissue models. Given the generally low irritation potential anticipated for this product category, endpoints extended beyond tissue viability (MTT) to include inflammatory markers (*e.g.*, cytokine expression). This design provided additional mechanistic insight into subtle irritation responses.

Within these studies, benchmark materials were used to support evaluation of test systems’ performance, including the ability of NAMs to detect formulation-driven differences in irritation potential (Roso et al., 2021). *In vitro* responses were interpreted relative to products with known or expected safety profiles, enabling calibration of assay sensitivity and dynamic range for low-to-moderate irritation effects (Martinez et al., 2014; Roso et al., 2021). In some cases, products not explicitly designated as benchmarks nevertheless functioned as such based on their consistent performance across studies. This strategy highlights the implicit use of benchmarking strategies in this space (Klausner et al., 2007; Koschier et al., 2011).

Benchmark-based approaches also facilitated comparative screening and rank-ordering of formulations (Martinez et al., 2014), particularly when assessing modifications to ingredient composition, such as changes in flavoring systems (Reed et al., 2023). For example, reconstructed oral tissue models have been used to evaluate the impact of formulation changes on irritation potential prior to clinical testing. This approach supports early-stage decision-making and reduces reliance on human studies (Reed et al., 2023). Similarly, benchmark-informed study designs have been applied to assess over-the-counter cough and cold formulations. In the experiments conducted by Walker et al., 2023, relevant marketed products were used as comparators to contextualize *in vitro* responses and to guide interpretation in the absence of fully validated regulatory frameworks.

In addition, benchmark strategies have been extended to more complex or personalized formulations. For example, the study by Hitzman et al., 2023 evaluated compounded oral rinses (*e.g.*, “magic mouthwash” formulations). The authors demonstrated the utility of reconstructed tissue models in assessing potential cytotoxic and inflammatory effects arising from combinations of otherwise well-characterized ingredients. In these contexts, benchmark materials with established safety profiles can support interpretation of assay outputs and identification of potential synergistic effects. However, further correlation with clinical data is often needed to strengthen confidence in the predictive capacity of the NAMs test systems.

Overall, benchmarks in oral care product testing serve to anchor NAM-based assay responses to known safety profiles. They enable both evaluation and calibration of test systems and facilitate comparative screening of formulation changes within a low-irritation domain.

## 4 Discussions

Although the use of benchmarks applies conceptually to all test systems, they became essential considerations to non-animal test systems. This is the case as NAMs generally require greater data interpretation for accurate toxicological predictions. In that regard, selection of benchmarks is a critical aspect on the decisions regarding safety assessment strategies. This aspect becomes of importance especially when test systems that do not have an established prediction model are selected.

The review and analysis of all publicly available resources referenced in our manuscript helped profiling the benchmark as a practical, applicable concept to take advantage of for the safety assessment of products from multiple industry branches. Our primary goal was to clarify the use of benchmarks in cosmetic and personal care industries, however along the way we came across examples from pharmaceutical industry which were introduced to support a comparative analysis.

### 4.1 Benchmarks profile

From a relevance perspective and to facilitate comparative data analysis, the benchmarks are expected to match the chemical/product type of the prototypes being evaluated and preferably the end use type. This aspect is of outmost importance when testing complex mixtures, formulations or products for which the relevant benchmark should be of similar composition, since the entire formulation (active and inactive components) has a significant effect upon the initial exposure kinetics of the active ingredients to the test system. For example, if the prototypes are toothpastes containing peroxide(s) of various concentrations, they should be compared with a product already on the market containing this particular ingredient. In this instance, the selection of the benchmark is in close connection with an active ingredient that might be responsible for an irritation effect. The selection criteria for benchmarks should take into consideration the product class, the type of product (leave on, rinse off, cosmetic or drug, medical device, etc.), the application site, exposure length, and composition.

The benchmarks should also have been previously characterized in the assay of choice and to have a toxicological profile supported by data (preferably human *in vivo* and/or clinical, animal, or *in vitro* from similar test systems). From a technical perspective, the benchmarks are also selected based on the understanding of the assay(s)’ limitations and the expertise of the user. There should also exist extensive market experience data to show that the benchmark’s potential impact relevant to the endpoint was deemed acceptable. Last but not least, ideally the benchmarks would have the same expected mechanism of action as the prototypes evaluated. Depending on the assay design and requirements, solubility, stability and purity of the benchmarks need to be established. Furthermore, their batch-to-batch variability should be tested with some regularity. Records of identity [Chemical Abstracts Service (CAS) number, batch number, purity, chemical structure, molecular weight, etc.], receipt, storage, preparation and use should be available to allow for a full reconstruction of the history and use of all benchmarks. The performance of the benchmark should also be tracked over time to demonstrate consistency in formulation.

Benchmarks should be non-hazardous (where possible) and be available from commercial sources without prohibitive costs and to carry an anticipated minimal risk of removal from the market (although this is not always a factor that is controllable). In instances where the benchmarks formulations are not available any longer or when changes in their formulations have been operated over the years, the users need to qualify a new benchmark and compare its performance in the assay with data generated using the retired benchmark. Depending on the industry needs, retirement of benchmarks may be or become a necessity, especially when a product line evolves to meet consumers’ needs or if a key ingredient is no longer available. Our analysis revealed that in some cases benchmarks are selected based on their known irritation profile to a specific target organ even though they are not of the exact product class as the prototypes. This is the case of N-9-containing gels used to investigate irritation potential of personal lubricants. This strategy is acceptable if a product of the same class is not available and/or capable to induce a positive response of the test system.

### 4.2 Benchmarks uses

From a company stewardship perspective, product benchmarking is critical to support improvement of products’ functional features (Patel et al., 2002). In that regard, individual companies use an *in vitro* assay with specific classes of chemicals that provide confidence for its predictive capacity relative to the material under evaluation (McNamee et al., 2009). Companies make frequent use of benchmarks to rank order their newer generations of prototypes compared to their own products or competitors and thus evaluate the competitiveness on the market.

Generally, the primary goal of the safety testing employing benchmark materials is based on evaluating the toxicity induced by the products and presented as % viability values or half-maximal effective concentration (EC_50_) or half-maximal inhibitory concentration (IC_50_) values. However, a recent study by Ayehunie et al. (2018) evaluated the use of a new *in vitro* test system reconstructed from human intestinal fibroblasts and enterocytes for predicting intestinal drug absorption and drug-drug interaction. The primary human cell-based organotypic small intestinal (SMI) microtissues allowed the expansion of the safety assessment besides the standard toxicity evaluation by examining drug-drug interactions using efflux transporter substrates and inhibitors. The permeability coefficients across the microtissues were determined for a panel of 11 benchmark drugs (Verapamil, Propanolol, Warfarin, Quinidine, Enalapril, Atenolol, Ranitidine, Cimetidine, Acebutanol, Erythromycin and Acyclovir) and were compared with known human absorption and Caco-2 permeability data. The SMI microtissues were able to discriminate between low and high permeability drugs and correlated better with human absorption data. The study indicated that SMI microtissues appear to be a useful preclinical tool for predicting drug bioavailability of orally administered drugs and expanded the use of benchmarks for this type of evaluations.

In regulatory toxicology, the benchmarks may be used for the submission of data on selected new substances to competent authorities. This would apply to substances for which the physical, chemical and other properties are known, and where it can be demonstrated that the use of the reference standards is toxicologically relevant. While benchmarks serve to “grade the response of the test system to the test item” (OECD, 2004), they do not necessarily provide enough information to assess the proper performance of the test system (like the positive control) (Ulrey et al., 2005; OECD, 2018). Thus, the definition of the reference item does not only include the use of an item utilized for the "absolute grading" of the response, but also for its use in "relative grading" [*i.e.*, the responsiveness of the test system (Seiler, 2005)]. These materials may play a key role in *in vitro* test methods by confirming that some aspect of the method is working as expected, such as verifying the performance of a plate reader (Petersen et al., 2021).

Benchmarks have been used to validate a new method [including *in silico* Skin Sensitization Risk Assessment – Integrated Chemical Environment (SARA-ICE)] as a replacement for the totally blind approach which currently exists, so that substances can be grouped into categories defined by the references standards (Reinke et al., 2025). Benchmarks can also be used to generate a prediction model for a method not yet validated or for the development and cross-validation of *in vitro* assays. Last but not least, reference standards could be used to investigate and calibrate new or existing assays by using data available in the public domain.

In designing *in vitro* assays without a prediction model, the concentration of the benchmarks and the exposure of the test system to them need to allow for small changes in the performance of the benchmarks to be measured between assays. In this instance, the test materials tested can have either greater or less activity than the benchmark to be evaluated (Cater and Harbell, 2013). Collation of these data over a period of time increases the reliability to support practical application of an assay/combination of assays tailored to a company’s product ingredients (McNamee et al., 2009).

**Fig. 1:**
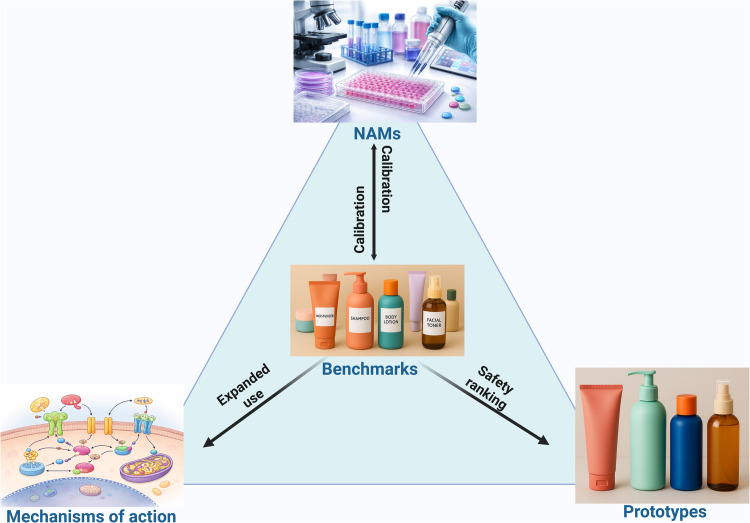
The central triangulated role of benchmarks for the evaluation of prototype ingredients or finished products. The NAMs and benchmarks are used in tandem to evaluate each other’s performance and fit for purpose. Furthermore, benchmarks can be used for the safety ranking of prototypes or to provide mechanistical insights regarding their efficacy potential for the intended end use.

The inclusion of benchmarks in the design of NAMs used for safety evaluations increases the reliance of the toxicologist on relevant products to compare the investigated prototypes to. The benchmarks are usually products on the market and thus have supporting data with clinical components, relevant to their use for human safety evaluations. In comparison, benchmarks are not typically utilized as routine concurrent controls for animal studies; therefore, this very specific approach embraced by the NAMs positions them as safety testing strategies more reliable compared to the *in vivo* methods. The existing NAMs have direct relevance for safety assessments as many are human-derived test systems. In contrast, even though the animal studies are considered the gold standard used when validating the counterpart NAMs, they are not human relevant and often incorrectly predict human responses. Furthermore, the NAMs have the capacity to provide organ-targeted mechanistic insights, while the animal tests provide an overall body or organ response but are rarely interrogated for molecular endpoints. Overall, the combination of NAMs and benchmarks provides a thorough strategy to be used for safety evaluations of a wide variety of personal care and cosmetic products (Fig. 1).

## 5 Conclusions and future directions

The advancement of the NAMs for the safety assessment of raw ingredients and finished formulations facilitated the use of benchmark materials for relevant comparative analyses, especially within the cosmetic and personal care industry. Even though this strategy is established for certain product lines or safety endpoints, as identified by our analysis of the 32 publicly available sources, we considered needed to write a review to summarize the learnings and understandings of the concept and its applications. Our research brought to light how various industries define and use the benchmarks. We determined that they are also used to support the development of new or optimization of established non-animal methods employed for safety assessments. The element that we consider the most critical is the criteria used for the selection of a benchmark for a defined goal. Specifically, one such criterion is the fit of the benchmark for the ultimate intended use and for the product category that is evaluated. Furthermore, the selection should be based on informed decisions supported by existing safety data. In this regard, the benchmarks can have a profile at either edge of the safety range (*e.g.*, irritating *vs.* non-irritating) to allow the compare-contrast evaluation of new prototypes. Another factor to consider when selecting benchmarks is their history of use in safety evaluations based on NAMs. This is especially important when such materials have been tested repeatedly and performance ranges have been established. We provided in our review a summary of strategies that can be used in the identification of the most relevant benchmark based on examples already reported in the literature. We hope that this review manuscript will support an expanded use of benchmarks materials for additional safety and efficacy endpoints, will advance their qualification for use in prototypes evaluations and also for the development of new NAMs.

## CRediT authorship contribution statement

**Huang Huang:** Conceptualization, Methodology, Resources, Visualization, Writing – review & editing. **Gertrude-Emilia Costin:** Conceptualization, Data curation, Investigation, Methodology, Resources, Supervision, Validation, Writing – original draft, Writing – review & editing.

## Declaration of competing interest

The authors declare that they have not competing interests.

## Data Availability

No data were used for the research described in the article.

## Appendix Supplementary materials

Image, application 1

Image, application 2

Image, application 3

Image, application 4

Image, application 5
