# Supplementary material for: Defining benchmarking in the context of safety assessment of personal care and cosmetic products using New Approach Methodologies
Source: NAM J. 2026 Jul 7;2:100111. doi: 10.1016/j.namjnl.2026.100111 (PMC13382591; doi:10.1016/j.namjnl.2026.100111)
Supplement: Supplementary file 1 [file mmc1.docx]

**Supplementary Table 1.** Raw ingredients used to calibrate the performance of *in vitro* test systems.

| **Raw ingredient class** | **Testing methodology** | | **Existing paired data** | **Data Summary** | **Reference** |
| --- | --- | --- | --- | --- | --- |
|  | **Test system** | **Endpoint** |  |  |  |
| - Biocides (3) - Chemical additive (1) - Colorant (1) - Conditioners (2) - Emollients (3) - Emulsifying agent (1) - Flavoring agent (1) - Preservatives (7) - Surfactants (22) - Amphoteric (5) - Anionic (7) - Cationic (4) - Non-ionic (6) - UV absorbers (2) | 3D human ocular tissue model (EpiOcular™ from MatTek Corporation) | Tissue viability (%) by MTT endpoint | Draize eye irritation | - Four eye irritation levels were considered based on the animal test: non irritating/minimal; mild; moderate; severe/extreme. - No ingredients were under-predicted by the *in vitro* assay (false negative). - Only 3 ingredients were predicted to be skin irritants by the *in vitro* assay compared to a non-irritant prediction *in vivo* (false positive). - Paired analysis of *in vitro* and *in vivo* data showed that 63% (27/43) of the ingredients were classified identically. - Assay performance improved to 95% (41/43) with the addition of samples over-predicted by a single irritation class. - This prediction model generated based on the paired *in vivo-in vitro* data is now grandfathered in protocols using the *in vitro* assay for prediction of eye irritation of raw ingredients and finished products. | Stern et al., 1998 |
| Ingredients specific to oral care products:   - Surfactant (SLS: 0.2%; 0.4%; 0.6%) - Dentifrice (NaF, NaF/PPI) - Mixtures of surfactant and poloxamer 407 | 3D human buccal mucosal tissue model (EpiOral™ from MatTek Corporation) | - Cytokine expression (IL-1α, IL-1β) - Tissue Viability (MTT) | Clinical data (referred to as comparative in the poster, but not included) | - These ingredients were specific to oral care product lines and were used to develop an *in vitro* methodology for determination of potential irritation of oral care excipients or finished products. - The increase in cytokine expression and decrease in tissue viability observed for the SLS treatments was consistent with the literature showing the toxicity of SLS to oral mucosal tissue. - The addition of poloxamer to SLS solutions demonstrated a decrease in toxicity associated with increasing cell viability and decreasing cytokine expression, which is also consistent with manufacturers’ data. The slight increase in cytokine expression and decrease in cell viability observed for the NaF/PPi dentifrice is consistent with human oral irritation data showing that PPi-containing dentifrices may cause slight irritation for select individuals. - In addition, low tissue viability and high cytokine expression levels were associated *in vitro* with experimental dentifrice formulations that failed the desquamation testing conducted as part of clinical studies. - The authors considered the test method used as correlative to human irritation results and as a reliable approach to estimate oral irritation level induced by various oral care products. | Bacca and Jewell-Motz, 2005 |
| - Alcohols (9) - Esters (6) - Ketones (5) - Miscellaneous substances (4) - Surfactants (12) | 3D human reconstructed corneal model (SkinEthic™ HCE from L’Oréal Research and Innovation Center) | - Tissue viability (%) by MTT endpoint - MCI - T_50_ cytotoxicity index | Draize eye irritation | - A proportion of 69.5% of the raw ingredients were correctly classified by the *in vitro* method. - The eye irritation induced by 7chemicals (4 surfactants, 2 esters and 1 alcohol) was under-predicted *in vitro* compared to the animal data, while the irritation of 4 ingredients (1 ester, 1 alcohol and 2 miscellaneous substances) was over-predicted. - If borderline ingredients were excluded from the analysis, only 5 ingredients were under-predicted and 3 over-predicted. - This paired data analysis was used to evaluate the predictive capacity of the test system for eye irritation that may be induced by raw ingredients, with the ultimate goal to replace animal testing for this endpoint. | Doucet et al., 2006 |
| - Chemicals (25) selected from the *in vivo* rabbit data published in the ECETOC Database No. 66 (ECETOC, 1995) for SIT - Bergamot oil (4 samples) for phototoxicity testing | - 3D human dermal tissue model (EpiDerm™ from MatTek Corporation) - 3T3 Balb/c fibroblasts | - 3T3 NRU phototoxicity test - EpiDerm™ phototoxicity test - SIT - tissue viability (%) by MTT endpoint (using both the 15-minute and 60-minute exposure times); OECD TG 439 - UV/Vis spectral analysis | - 4 h HPT - Draize skin irritation - Human photo-patch test | - Several chemicals reported to be skin irritants in the rabbit test were found to induce no effects in humans. - The 3D skin model-based assays and the human patch tests provided concordant results particularly in the case of non-irritating and non-phototoxic substances. This platform is a useful tool for predicting human skin irritation and phototoxicity hazard, particularly for consideration of initial concentration for confirmatory human patch tests to prove the safety of raw ingredients and finished products. The 15-minute exposure under-predicted 1 borderline human irritant (1-bromohexane) and classified 2 substances as false positive, while the 60-minute protocol predicted all human irritants as such, however provided a couple of false positive results, thus being more conservative. - The 4 h HPT data should represent the gold standard to detect acute skin irritation potentials when approaching validation of alternative methods. - The benchmarks selected had well established safety data generated in animals and human studies, thus providing data for paired analysis and also for the re-evaluation of the *in vitro* test. | Jirova et al., 2007 |
| Surfactants (16):   - Amphotheric (2) - Anionic (6) - Non-ionic (8) | 3D human dermal tissue model (EpiDerm™ from MatTek Corporation) | - Tissue viability (%) by MTT endpoint - Cytokine expression (IL-1α) | NA | - The selected raw ingredients class (surfactants) is representative to the cosmetic products investigated in this manuscript. They were used to evaluate the sensitivity of the test system and of the IL-1α endpoint in predicting human skin irritation and to calibrate the performance of the test system and of the method. | Walters et al., 2016 |
| Surfactants:   - Benzalkonium chloride - Cetylpyridinium - Lauryl betaine - Lautrimonium chloride - Sodium lauryl sulfate | 3D human epidermis model (Labcyte EPI-MODEL from J-TEC) | - Tissue viability (%) by MTT endpoint - CMC - Cytokine expression (IL-1α) | 24 h HPT data | - Of the 5 surfactants tested, sodium lauryl sulfate, lauryl betaine and benzalkonium chloride were previously reported to be false negatives when evaluated *in vitro* for skin irritation. - The *in vitro* and clinical results were consistent when using a margin of two. - The raw ingredients were used in this case to calibrate the concentrations to be considered for the design of the *in vitro* method in order to accurately predict human clinical data for skin irritation. | Sugiyama et al., 2019 |
| - 4-Aminobenzoic acid (cosmetic) - Anthracene (pharmaceutical) - Bergamot oil regular and Kosher (cosmetic and food industry) - Chlorpromazine hydrochloride (pharmaceutical) - Cinnamaldehyde (cosmetic and food industry) | 3D human dermal tissue model (EpiDerm™ from MatTek Corporation) | - Tissue viability (%) by MTT endpoint (cytotoxicity) - Phototoxicity | Previously conducted *in vitro* studies (Liebsch et al., 1997; Liebsch et al., 1999; Jones et al., 2003) | - The cytotoxicity and phototoxicity results obtained for the reference chemicals were as expected and comparable with data obtained in previously reported studies. Thus, they were used to properly calibrate the prediction of the phototoxic effect of new materials. - Three TiO_2_ nanoparticle sheets (Aeroxide P25, Eusolex T-2000 and TIG-800) were tested at several concentrations. - None of the TiO_2_ nanoparticle sheets investigated induced phototoxicty or cytotoxicity up to the highest concentration tested. An explanation would be that the tested nanoparticles do not penetrate deep enough into the viable cells of the reconstructed epidermis to cause cytotoxicity or phototoxicity. | Líšková et al., 2020 |
| 42 substances relevant to cosmetics and quasi-drugs | 3D human dermal tissue model (EpiDerm™ from MatTek Corporation) | SIT - tissue viability (%); OECD TG 439 | - 24 h HPT data - Draize skin irritation - Human cumulative skin irritation test | - A workflow was proposed, using the physicochemical properties or existing information as the first step, followed by SIT. Several false negative results were obtained as compared to the HPT. Therefore, if a test substance is negative in SIT, human skin irritation tests are still needed for confirmation. - The SIT and the human skin tests can be used to evaluate test substances that cause weak or no irritation and are categorized as “harmless ingredients”, thereby avoiding animal testing. - The chemicals were considered for benchmarking purposes, in order to calibrate the performance of the *in vitro* assay when compared to paired animal and human data. | Kojima et al., 2021 |

3D, three-dimensional (referring usually to tissue models); CMC, Critical Micellar Concentration; ECETOC, European Centre for Ecotoxicology and Toxicology of Chemicals; HPT, Human Patch Test; IL, Interleukin; MCI, Mean Cytotoxicity Index; MTT, 3-(4,5-dimethylthiazol-2-yl)-2,5-diphenyltetrazolium bromide; NA, Not Applicable; NaF, sodium fluoride; OECD, Organisation for Economic Co-operation and Development; SIT, Skin Irritation Test; PPI, pyrophosphate; SLS, Sodium Lauryl Sulfate; T_50_, the time required for a 50% reduction in MTT metabolism in the treated cells; TG, Test Guideline; UV, ultraviolet; Vis, Visible (light)

Note: The references are presented in chronological order and alphabetically within the same year (where applicable).

References:

ECETOC. Technical report no. 66. Skin irritation and corrosion: reference chemicals data bank. 1995. Available at: <https://www.ecetoc.org/wp-content/uploads/2014/08/ECETOC-TR-066.pdf>.

Jones, P.A., King, A.V., Earl, L.K., Lawrence, R.S. An assessment of the phototoxic hazard of a personal product ingredient using *in vitro* assays. Toxicol In Vitro. 2003 Aug;17(4):471-480. doi: 10.1016/s0887-2333(03)00048-1. PMID: 12849731.

Liebsch, M., Barrabas, C., Traue, D., Spielmann, H. Development of a new *in vitro* test for dermal phototoxicity using a model of reconstituted human epidermis. ALTEX. 1997;14(4):165-174. English. PMID: 11178502.

Liebsch, M., Traue, D., Barrabas, C. *et al*. (1999). Prevalidation of the EpiDerm phototoxicity test. In D. Clark, S. Lisansky and R. Macmillan (eds.), *Alternatives to Animal Testing II: Proceedings of the second international scientific conference organized by the European Cosmetic Industry, Brussels, Belgium* (160-166). Newbury, UK: CPL Press.
