# Supplementary material for: Defining benchmarking in the context of safety assessment of personal care and cosmetic products using New Approach Methodologies
Source: NAM J. 2026 Jul 7;2:100111. doi: 10.1016/j.namjnl.2026.100111 (PMC13382591; doi:10.1016/j.namjnl.2026.100111)
Supplement: Supplementary file 2 [file mmc2.docx]

**Supplementary Table 2.** Benchmarks used for safety assessment of home cleaning products.

| **Type of products tested** | **Benchmark(s) tested** | **Testing methodology** | | **Existing paired data** | **Data Summary** | **Reference** |
| --- | --- | --- | --- | --- | --- | --- |
|  |  | **Test system** | **Endpoint** |  |  |  |
| Air fresheners | Thickened fragrance formulation (complex fragrance mixture) | BCOP | Eye irritation  (IVIS) | Draize eye irritation | - Reproducibility of the assay over time was evaluated using a longitudinal analysis of the benchmark material which was used as comparative standard for evaluating prototype air fresheners (tested undiluted). - The assay was capable to distinguish minor changes in benchmark preparation (p<0.05). - Since fragrance mixtures frequently contain >100 components of widely varying volatility, it was hypothesized that the continuous interactions occurring in the mixture itself may contribute to the variability in the data over time. The fragrance was a hydrophobic, solvent-based mixture used in an aqueous environment and may not have been completely removed from corneas, resulting in more variable results. - The data obtained emphasize the need to test the performance of an established benchmark over time, especially when prepared multiple times over the span of several years (over 60 benchmark values obtained over a 5-year period while being reformulated 3 times). - The benchmark (predicted to be moderately irritating *in vivo*) was also used to assess the reproducibility of the test system over time. | Ippolito et al., 1999 |
| Aerosol fragrances: 44 formulations based on 7 fragrance systems | Aerosol formulations (fragranced) in the form of a gel and aerosol used to normalize their respective product category | BCOP | - Eye irritation (IVIS) - Histology | NA | - The benchmark selected was used to determine the optimal exposure time course for the assay, while the products with known irritation potential were used to establish a prediction model specific for the product line. - The established eye irritation profile of the benchmark provided a standard for determining an acceptable level of eye irritation potential for the product category investigated. - The experiments recommended a testing protocol for a surfactant-based product to use a 25% (v/v) aqueous dilution, a 30-minute exposure, concurrent testing of the benchmark control and using permeability values for the evaluation of eye irritation potential in addition to histopathological evaluation. | Cuellar et al., 2002 |
| Laundry detergents:   - Liquid (17) diluted to 25% - Granular (11) diluted to 10% | - Commercially marketed liquid laundry detergent selected as an Ultra Liquid Laundry Detergent - Commercially marketed granular laundry detergent selected as an Ultra Granular Laundry Detergent | BCOP | - Eye irritation (IVIS) - Histology | - Draize eye irritation - Market history data | - Review of the histological evaluation showed that the benchmark formulas induced damage to the epithelium that extended through the squamous cell layer and partially into the wing cell layer. These observations were consistent to the OD_490_ scores obtained and reflected moderate loss in epithelial cell layers. - Both benchmarks were classified *in vivo* as a moderate eye irritant. The permeability values for the benchmarks fell within the middle of the dynamic range. - The experiments focused on the selection of well-characterized benchmarks and the development of decision criteria that allow data interpretation on new materials. - Upper-end benchmark formulas were tested for each product class. They set a known upper range for acceptable irritation for the product class. - Each benchmark was used to determine the standard conditions for its product class in the assay. | Cater and Harbell, 2013 |
| - Air fresheners - Home cleaning products | Selected to be specific to each product line investigated | BCOP | - Eye irritation (IVIS) - Histology | NP | - Benchmarks were used to evaluate specific product lines. They were selected to facilitate comparative interpretation of the results. - The poster emphasizes the importance of using benchmarks relevant to the product line the prototypes belong to. | Swanson et al., 2016 |
| Mild laundry detergents (DPD was the target laundry investigated) | Existing mild laundry detergents (11) | - 3D epidermal skin model (EpiDerm™ model from MatTek Corporation) - Superficial SC layers collected from healthy volunteers | - Inflammatory response: Epiderm™ model, tissue viability by MTT and IL-1α endpoints) - Overall SC damage (*ex vivo* method corneosurfametry) - Protein denaturation: Zein solubility assay (*in vitro*) | NA | - The objective of the experiments was to create an especially mild laundry detergent formulation (DPD formulations) and to concurrently compare its mildness profile with existing mild laundry products on the market. - The DMI was derived from the three endpoints used to create a comprehensive examination of the toxicological profile of the detergents. | Fowler et al., 2017 |

3D, three-dimensional (referring usually to tissue models); BCOP, Bovine Corneal Opacity and Permeability; DMI, Detergent Mildness Index; DPD, Development in partnership with dermatologists; IL, Interleukin; IVIS, In Vitro Irritation Score; MTT, 3-(4,5-dimethylthiazol-2-yl)-2,5-diphenyltetrazolium bromide; NA, Not Applicable; NP, Not Provided; OD, Optical Density; SC, *Stratum Corneum*

Note: The references are presented in chronological order and alphabetically within the same year (where applicable).
