# Supplementary material for: Defining benchmarking in the context of safety assessment of personal care and cosmetic products using New Approach Methodologies
Source: NAM J. 2026 Jul 7;2:100111. doi: 10.1016/j.namjnl.2026.100111 (PMC13382591; doi:10.1016/j.namjnl.2026.100111)
Supplement: Supplementary file 3 [file mmc3.docx]

**Supplementary Table 3.** Benchmarks used for safety assessment of personal care products (surfactant-based, hair products, skin care).

| **Benchmark(s) tested** | **Testing methodology** | | **Existing paired data** | **Data Summary** | **Reference** |
| --- | --- | --- | --- | --- | --- |
|  | **Test system** | **Endpoint** |  |  |  |
| **Surfactant-based products: shampoos, cleansers, soaps, etc.** | | | | | |
| Liquid hand soap (25%) | BCOP | - Eye Irritation (IVIS –-primarily permeability values - Histology | - Draize eye irritation (for the benchmark and products) - Market history data (benchmark) | - Four commercial and 1 prototype body wash developed specifically for children or as mild bath products were tested. - The benchmark selected was used to determine the optimal exposure time course (established at 30 minutes), while the products with known irritation potential were used to establish a prediction model specific for the product line. - The established eye irritation profile of the benchmark provided a standard for determining an acceptable level of eye irritation potential for the product category investigated. - The *in vitro* assay was considered to allow for formula optimization of mild bath products prior to investing in human eye sting tests since BCOP showed promise as a prediction model for relative ranking of conjunctival erythema responses in the human eye. | Cater et al., 2001 |
| ***Chemicals were used as benchmarks/references to calibrate the test system (see Table 1)*** | 3D human reconstructed corneal model (SkinEthic™ HCE from L’Oréal Research and Innovation Center) | - MCI - T_50_ cytotoxicity index - Tissue viability (%) by MTT endpoint | Draize eye irritation | - A total number of 16 bath and cleansing products were evaluated using this method as 5% dilutions prepared in saline. Other product categories have been included (**see Face and body lotions section of this table**). - Of the products tested (all categories), 86% were correctly classified by the *in vitro* method. Only 1 cleanser was under-predicted, while 2 shower gels and 1 shower foam were over-predicted when compared with *in vivo* data. - Even though not discussed in the manuscript, given the high paired *in vivo-in vitro* data correlation rate for the finished products tested, it might be conceivable to consider some of these products for qualification as benchmarks.^^^ | Doucet et al., 2006 |
| Two shampoos based on TriBlend 3 containing Sodium Laureth Sulfate, Sodium Lauryl Sulfate and Cocamidopropyl Betaine | 3D human reconstructed ocular tissue model (EpiOcular™ from MatTek Corporation) | % tissue viability (MTT) | Consumer adverse experience data | - A total of 28 shampoos, 13 facial cleansers and 7 body washes were tested. - A total of 5 surfactant systems (based on 2 or 3 surfactants mixed together) used in shampoos were investigated. Only 2 shampoos were tested as 10% dilutions, while the rest were tested as 2% dilutions. - Even though both were based on TriBlend 3, Benchmark #1 had an ET_50_ value of >4 h, while Benchmark #2 had an ET_50_ value of 1.85 h. The combo of two different responses based on benchmarks allowed a more accurate comparison and interpretation of the data obtained for the products tested. - The irritancy potential for most of the prototype shampoos fell in the minimal to no irritation range and showed similar or less cytotoxic responses compared to the reference materials. An existing prediction model based on *in vivo* results (Kay and Calandra, 1962), and *in vitro* data from MatTek Corporation and McCain et al., 2002 was used for the evaluation and interpretation of the results. - The facial cleansers were based on surfactant systems composed of 1, 2, 3, or 4 surfactants. - Most of the cleansers were tested as 2% dilutions, while 3 were tested as 10% dilutions, and the ET_50_ values were indirectly correlated with the concentration (a shorter ET_50_ value pertaining to a higher concentration). - Most of the body washes were tested as 10% dilutions, and 2 of them as 2% dilutions and were predicted to be within the mild to no irritation range. - The *in vitro* data correlated well with the consumer experience information, thus making the EpiOcular™ test system very useful for deciding whether a product moves forward from prototype status to the market. | Vavilikolanu et al., 2008 |
| Two benchmark cleansers with known clinical skin irritation potential (Mild Cleanser 1 was tested 32 times, while Mild Cleanser 2 was tested 35 times). They contained a mixture of anionic, amphotheric and non-ionic surfactants | 3D human dermal tissue model (EpiDerm™ from MatTek Corporation) | - Cytokine expression (IL-1α) - Tissue viability (%) by MTT endpoint | Clinical data (exaggerated patch test with TEWL endpoint) | - A total of 46 commercial surfactant-based cleansers and 162 other prototype surfactant-based cleansers (non-commercialized formulations) were evaluated. - The 2 representative benchmarks with known clinical skin irritation potential were qualified through repeated testing for use as references for the skin irritation evaluation of formulations containing new surfactant ingredients. - Although the viability assay showed no difference between the two benchmarks, there was a clear difference in IL-1α production between them, indicating that the sensitivity of this endpoint was greater than that of the MTT viability endpoint. The data also indicated that the second mild cleanser had a slightly greater irritation potential than the first cleanser based on the cytokine expression endpoint. - The use of the combo of 2 benchmarks with different skin irritation profile facilitated the evaluation of new surfactants. - A total of 28 cleansers were tested using the *in vitro* test system and subsequently in clinical studies and showed a predictive correlation between the two methods. | Walters et al., 2016 |
| - Concentrated nitric acid (strong/severe, possibly corrosive, ET_50_ value <0.5 h) - 1% Sodium Dodecyl Sulfate (moderate, ET_50_ value 0.5-4 h) - 1% Triton X-100 (moderate to mild, ET_50_ value 4-12 h) - Baby shampoo (very mild, ET_50_ value 12-24 h) - 10% Tween 20 (non-irritating, ET_50_ value 24 h) | 3D human reconstructed skin tissue model (EpiDerm™ from Mattek Corporation) | % tissue viability (MTT) | NA | - A total of 34 shampoos, 1 cleanser and 4 soaps were tested. - The aim of this study was to evaluate the skin irritation potentials of topically applied cosmetic products which were marketed in Turkey between 2015 and 2017 by using a reconstructed skin model as test system. - Even though this screening (time-to-toxicity) assay does not have a prediction model, the authors used the correlations provided by the tissues manufacturer to assigning verbal descriptors for expected *in vivo* irritation based on the ET_50_ values obtained using the EpiDerm™ tissue model.* - Based on these correlations, 11 shampoos were anticipated to be moderate to mild irritant to the skin, 22 moderate and 1 strong/severe. The cleanser was anticipated to have moderate skin irritation potential and the soaps were anticipated to be non-irritating. - The authors considered the chemicals/finished formula included in the correlation as benchmarks; except for the nitric acid, the rest of the chemicals were surfactants or surfactant-based (the baby shampoo), thus making them relevant benchmarks for the data interpretation for the product category investigated.* | Kose et al., 2018 |
| NA (however, tested products might be considered for qualification) | 3D human reconstructed skin tissue model (EpiSkin from L’Oréal Research and Innovation Center) | - Cytokine expression (IL-1α) - Histology - Tissue viability (%) by MTT endpoint | Draize skin irritation data | - A total of 80 cosmetic and personal care products were tested, 11 of which were cleansing foams. - The test methodology was used to determine the skin irritation induced by resident and cleaning products and identified the exposure time of 18 h as relevant for leave-on products. - For cleansing products, a 1 h exposure with 10% dilution for the products was suggested. - Compared with the animal data, the *in vitro* test system was found to objectively respond with reliability to skin irritation induced by finished cosmetic products. - Even though not discussed in the manuscript, given the high correlation rate with animal data for the finished products tested, it might be conceivable to consider some of these products for qualification as benchmarks. | Ma et al., 2021 |
| NA (however, tested products might be considered for qualification) | - 3D human reconstructed skin tissue model (EpiSkin® from L’Oréal Research and Innovation Center) - 3D human reconstructed corneal tissue model (SkinEthic™ HCE from L’Oréal Research and Innovation Center) | - Cytokine expression (IL-1α) - HET-CAM (eye irritation) - Tissue viability (%) by MTT endpoint | Consumer research data (2 weeks) | - A total of 8 baby cleaning products were tested within a larger group of products currently available on the market. The rest of the products were 2 types of baby wipes, 2 body oils and 2 body creams. - The results obtained using the 3D test systems and the consumer research correlated in showing no irritation induced by the products tested. However, the HET-CAM test system predicted some of the products to be irritating to the eye: all 8 cleaning products were predicted to be irritating (2 moderate irritants and 6 slightly irritating). These results might be related to the surfactant load in these products. - The authors proposed a safety testing strategy to start with HET-CAM in order to assess if the products might be classified as slight irritants and then continue by the use of 3D test systems. Consumer research or clinical trials might be considered in the end for more valid proof. - Although no products tested were referred to as benchmarks, their safety is implied by being available to the end user and might be considered for qualification as reference materials. | Wang et al., 2021 |
| - Benzyl alcohol - HICC - IPBC - MCI/MI - MDBGN - MI - Phenoxyethanol - Propyl gallate - Propyl paraben - Sodium benzoate | *In silico* platform (SARA) for skin sensitization | Data included in the tool were generated from:   - DPRA - KeratinoSens™ - h-CLAT - U-SENS | - HRIPT - LLNA | - The surfactant-based benchmarks were representative of liquid hand soaps, shampoos and shower gels for all ingredients except for propyl gallate and HICC, and of high, low and unclassifiable induction risk categories. - The authors investigated whether the use of safety factors may be circumvented altogether in NGRA by using benchmark exposure information to derive empirical support that an exposure is low risk and can be considered safe.^#^ - Margins-of-exposure (potency estimate to consumer exposure level ratio) were regressed against the benchmark risk classifications, enabling derivation of a risk metric defined as the probability that an exposure is low risk. - Benchmark exposures pertaining to use of consumer products were integrated with clinical data to support a high/low risk categorization for skin sensitization.^#^ - A benchmark exposure was classified as high risk when there was strong evidence in the literature that a historical exposure to the contact allergen in a given product type led to a significant burden of contact allergy in the population. Low risk was assigned when despite a long-standing history of exposure to the benchmark the evidence suggests that there is not a significant burden of contact allergy in the population.^#^ | Reynolds et al., 2022 |
| **Hair products** | | | | | |
| Reference material based on a dual 8 quaternary system (quaternium-18 and quaternium-80) | - 3D human reconstructed ocular tissue model (EpiOcular™ from MatTek Corporation) | % tissue viability (MTT) | Consumer experience data | - A total of 27 rinse-off hair conditioners based on either single or dual quaternary ammonium systems were tested undiluted. - The vast majority of the products showed insignificant relative toxicity up to 24 h exposure *in vitro*, indicating that they were not anticipated to be irritating to the eyes. Furthermore, the effectiveness of the test system has been assessed by comparing the *in vitro* results with consumer experience information. - The benchmark was used as an added assessment tool. It was a material with similar chemistry to the products evaluated and had well established safety assessment data. The exposure time response of the products with similar formulations could be directly compared to the exposure time response of the reference material to determine if the prototypes would likely cause greater, lesser or equal level of irritation. Thus, the products can be classified into simple categories of “acceptable/unacceptable” based on the limit(s) set by the reference material(s). | Vavilikolanu et al., 2007 |
| Ethanol (6%, 55%, and 80%) | - 3D human reconstructed ocular tissue model (EpiOcular™ from MatTek Corporation) - 3D human reconstructed skin tissue model (EpiDerm™ from Mattek Corporation) | % tissue viability (MTT) | - Consumer adverse experience data - RIPT | - A total of 8 VOC-containing products (hair sprays, mousses, etc.) were tested undiluted. - In this project, the effects of a lower than typical dose of the product exposed to the EpiOcular™ and EpiDerm™ tissue models was examined. This was an initial evaluation of a procedure modification to lessen the over-prediction of irritancy commonly observed in these assays for products with significant VOC concentrations such as hair sprays. A dose volume of 30 μL was selected, which minimized variability of results among tissues’ responses within a treatment group. Also, in modeling consumer exposure scenarios, a small volume exposure is more realistic as compared to an “infinite” dose”, modeled by the standard 100 μL dose volume covering the topical surface of the tissue completely for the full duration of the exposure time. These predictions correlated well with the lack of adverse consumer experiences and the RIPT’s no irritation potential. - The benchmark’s concentrations selected provided the reference cytotoxicity responses caused by VOCs at typical low, intermediate, and high concentrations. - This manuscript brings forward a strategy to investigate the ocular and dermal irritation that might be induced by VOC-based products using the same set of benchmarks which also were used to calibrate the test system’s performance and to align it with human exposure data. | Vavilikolanu et al., 2009 |
| ***Same as for Surfactant-based products section, same author*** | 3D human reconstructed skin tissue model (EpiDerm™ from Mattek Corporation) | % tissue viability (MTT) | NA | - A total of 7 non-surfactant-based hair products were tested (2 hair creams, 2 herbal hair oils, 2 hair lotions and 1 hair serum). - Based on the guidance mentioned in the Surfactant-based section above (marked *), 1 hair cream was anticipated to be very mild, while the other to be moderate. Of the 3 hair oils, 2 were anticipated to be non-irritating, while 1 to be a strong irritant to the skin. The hair serum and hair lotion were anticipated to be moderate to mild skin irritants. - ***Note(s) marked “*” from Surfactant-based products section, same author apply for this category.*** | Kose et al., 2018 |
| **Face and body lotions** | | | | | |
| ***Chemicals were used as benchmarks/references to calibrate the test system (see Table 1)*** | 3D human reconstructed corneal tissue model (SkinEthic™ HCE from L’Oréal Research and Innovation Center) | - MCI - Tissue viability (%) by MTT endpoint - T_50_ cytotoxicity index | Draize eye irritation | - A total number of 36 skin and sun care products were evaluated undiluted using this method. - Of the products tested (all categories), 86% were correctly classified by the *in vitro* method. Of the skin and sun care category, 4 were over-predicted in their eye irritation potential *in vitro vs.* the Draize test and among those, 3 belonged to those formerly described for which the MMAS value was questionable (their MMAS values were not representative of their respective individual Draize test scores). - ***Notes marked”^^^” from Surfactant-based products section, same author apply for this category.*** | Doucet et al., 2006 |
| FDA benchmark threshold of 3.4ppm for leave-on body lotions (U.S. FDA, 1996) | - 3D human reconstructed skin tissue model (EpiDerm™ from Mattek Corporation) - BCOP - CAMVA | - Eye irritation (IVIS) - RC_50_ - Tissue viability (%) by MTT endpoint | - Clinical human skin irritation data - Post-market surveillance data | - A total of 19 BTC-containing body lotions were evaluated by using a combination of *in vitro* testing, clinical studies, monitored home usage tests, and post-marketing surveillance data. - The EU restricted the use of BTC as preservative to ≤ 0.1%, however this class of compounds has been safely used for many years at concentrations ≤5% in cosmetic products. The data included in this manuscript showed evidence that BTC is safe when formulated appropriately in finished whole-body, leave-on lotions in the concentration range of 1-5%. - The study did not include a benchmark *per se*, however FDA’s guidance on BTC was used to evaluate the products as follows: the relevance of the negative results obtained in the *in vitro*, clinical patch test, and consumer usage tests for BTC-containing leave-on body lotions was confirmed by extensive post-marketing surveillance data. Post-marketing data for 5 of these body lotions during a 5-year period (2006–2011) showed an overall rate of only 0.69 undesirable effects (skin irritation) per million shipped consumer units, which was remarkably lower than the FDA benchmark threshold of 3.4 ppm for leave-on body lotions (U.S. FDA, 1996). - Even though not discussed in the manuscript, given the high correlation rate of the data obtained for the finished products when tested *in vitro* and in clinical setting, it might be conceivable to consider some of these products for qualification as benchmarks. | Cameron et al., 2013 |
| ***Same as for Surfactant-based products section, same author*** | 3D human reconstructed skin tissue model (EpiDerm™ from Mattek Corporation) | % tissue viability (MTT) | NA | - A total of 5 non-surfactant-based skin care products were tested (3 creams and 2 masks). - Based on the guidance mentioned in the **Surfactant-based section above (marked *)**, 2 creams were anticipated to be non-irritating to the skin, while 1 to be moderate to mild. The masks were estimated to be very mild irritants. - ***Note(s) marked “*” from Surfactant-based products section, same author apply for this category.*** | Kose et al., 2018 |
| NA (however, tested products might be considered for qualification) | 3D human reconstructed skin tissue model (EpiSkin® from L’Oréal Research and Innovation Center) | - Cytokine expression (IL-1α) - Histology - Tissue viability (%) by MTT endpoint | Draize skin irritation data | - A total of 80 cosmetic and personal care products were tested, 19 of which were creams, 8 emulsions, and 13 lotions. - The test methodology was used to determine the skin irritation from resident and cleaning products and identified the exposure time of 18 h as relevant for leave-on products. - Compared with the animal data, the *in vitro* test system was found to objectively respond with reliability to skin irritation induced by finished cosmetic products. - Even though not discussed in the manuscript, given the high correlation rate of the data obtained for the finished products when tested *in vitro* and *in vivo* setting, it might be conceivable to consider some of these products for qualification as benchmarks. | Ma et al., 2021 |
| ***Same as for Surfactant-based products section, same author*** | *In silico* platform (SARA) for skin sensitization | Data included in the tool were generated from:   - DPRA - KeratinoSens™ - h-CLAT - U-SENS | - HRIPT - LLNA | - Based on clinical evidence and overall risk ranking, the authors selected several benchmarks for each of the ingredients investigated, as follows: - Benzyl alcohol: 1 face cream and 1 body lotion, both at low induction risk (2 concentrations) - IPBC: 1 face cream of low induction risk (1 concentration) - MDBGN: 1 face cream and 1 body lotion, both at high induction risk (1 concentration) - MCI/MI: 1 face cream of high induction risk (2 concentrations) and 1 body lotion (high risk, 2 concentrations) - MI: 1 face cream and 1 body lotion, both at high induction risk (1 concentration) - Phenoxyethanol: 1 face cream and 1 body lotion, both at low induction risk (1 concentration) - Propyl paraben: 1 face cream and 1 body lotion, both at low induction risk (2 concentrations) - Sodium benzoate: 1 face cream and 1 body lotion, both at low induction risk (1 concentration)   The ingredients were used to train the *in silico* platform in its capacity to predict skin sensitizers.   - ***Note(s) marked “*#” *from Surfactant-based products section, same author apply for this category.*** | Reynolds et., 2022 |

3D, three-dimensional (referring usually to tissue models); BCOP, Bovine Corneal Opacity and Permeability; BTC, behentrimonium chloride; CAMVA, Chorioallantoic Membrane Vascular Assay; DPRA, Direct Peptide Reactivity Assay; EU, European Union; ET_50_, Effective Time necessary to reduce the viability of tissues to 50% of the viability of the negative control-treated tissues; FDA, Food and Drug Administration; h-CLAT, human Cell Line Activation Test; HET-CAM, Hen’s Egg Test – Chorioallantoic Membrane (Test Method); HICC, Hydroxyisohexyl 3-cyclohexane carboxaldehyde; HRIPT, Human Repeated Insult Patch Test; IL, Interleukin; IPBC, iodopropynylbutylcarbamate; IVIS, In Vitro Irritation Score; LLNA, Local Lymph Node Assay; MCI, Mean Cytotoxicity Index; MCI/MI, methylchloroisothiazolinone/methylisothiazolinone; MDBGN, methyldibromoglutaronitrile; MI, methylisothiazolinone; MMAS, Modified Maximum Average Score; MTT, 3-(4,5-dimethylthiazol-2-yl)-2,5-diphenyltetrazolium bromide; NA, Not Applicable; NGRA, New Generation Risk Assessment; RC_50_, the concentration at which 50% of the eggs exhibited positive responses (vascular hemorrhaging, capillary injection, or vascular lysis); SARA, Skin Allergy Risk Assessment; T_50_, the time required for a 50% reduction in MTT metabolism in the treated cells; TEWL, Trans-Epidermal Water Loss; VOC, Volatile Organic Compound

Note: The references are presented in chronological order and alphabetically within the same year (where applicable).

References:

Kay, J.H., Calandra, J.C. Interpretation of eye irritation tests. J Soc Cosmetic Chem. 1962;13:281-289.

McCain, N.E., Binetti, R.R., Gettings, S.D., Jones, B.C. Assessment of ocular irritation ranges of market-leading cosmetic and personal-care products using an *in vitro* tissue equivalent. The Toxicologist. 2002;66:243. Abstract available at: <https://www.toxicology.org/pubs/docs/Tox/2002Tox.pdf>.

U.S. FDA (1996). Voluntary filing of cosmetic product experiences (21CFR730). Code of Federal Regulations, vol. 7(Part 730). Food and Drug Administration, Department of Health and Human Services, Washington, DC, pp. 198–200. (Title 21, Revised 01.04.96).
