# Supplementary material for: Defining benchmarking in the context of safety assessment of personal care and cosmetic products using New Approach Methodologies
Source: NAM J. 2026 Jul 7;2:100111. doi: 10.1016/j.namjnl.2026.100111 (PMC13382591; doi:10.1016/j.namjnl.2026.100111)
Supplement: Supplementary file 4 [file mmc4.docx]

**Supplementary Table 4.** Benchmarks used for safety assessment of intimate care products.

| **Type of products tested** | **Benchmark(s) tested** | **Testing methodology** | | **Existing paired data** | **Data Summary** | **Reference** |
| --- | --- | --- | --- | --- | --- | --- |
|  |  | **Test system** | **Endpoint** |  |  |  |
| Vaginal care products:   - Anti-fungal agent (3) - Anti-itch cream (4) - Douche (2) - Feminine wash (3) - Lubricant (2) - Spermicide (4) | - KY Jelly - KY Jelly spiked with N-9 (0.1% and 2%) - N-9 (0.1%) | 3D organotypic vaginal - ectocervical tissue model (EpiVaginal™ - VEC from MatTek Corporation) | - Characterization of the tissue model by: histology, PAS staining, immunohistochemical analysis of cytokeratin, TEM - Cytokine expression: IL-1α and 1L-1β, TNF-α) - Histology - Tissue viability (%) by MTT endpoint | NA | - Based on the ET_50_ values, the irritation potential of the products evaluated could be rank-ordered as follows: feminine washes> spermicides > anti-itch creams > anti-fungal agents, douche, and lubricant. - The loss in tissue viability paralleled the structural damage was observed in the following order for the benchmarks: 2% N-9 > 0.1% N-9 > KY Jelly. - Histological damage of the tissues was inversely related to the ET_50_ values (short ET_50_ values were associated with pronounced histological damage and vice versa). - The combination of ET_50_ values and histological evaluation could discriminate between irritation potential of products within the same category. - IL-1α and IL-1β concentrations increased as structural damage also increased, while TNF-α release decreased as structural damage and loss in tissue viability increased. - Gynol II is a spermicide and thus is a directly comparable benchmark to the products within the same class that were evaluated; however, it is often included in studies investigating personal lubricants (grandfathered) as a control for irritation even though it has a different composition type and safety profile. - The pair of benchmarks covered both ends of the irritation spectrum and thus supported the rank-ordering of irritation potential induced to the tissues by the products investigated. | Ayehunie et al., 2006 |
| OTC personal lubricants:   - Aqueous-based (10) - Lipid-based (2) - Silicone-based (2) | Spermicide containing N-9 (2%) | - Colorectal epithelial cell line (Caco-2) - Endometrial epithelial cell line (HEC-1-A) - HeLa epithelial cell line stably transfected with an HIV-1 long terminal repeat linked to β-galactosidase and luciferase genes (TZB-bl) - *L. jensenii* - Normal human ectocervical and colorectal tissues from pre-menopausal women | - Anti-HIV-1 activity (TZM-bl and explants infected with HIV) - Cell viability (CellTiter-Glo™) - Normal vaginal flora testing - Tissue viability (MTT) | NA | - The products tested were marketed; their safety evaluation was assumed to have been conducted given the access of the consumers to the products (commercially available). - Gynol II contains 2% N-9 and was used as a toxicity control in the experiments given its established irritating profile for human exposure. Even though water-based, Gynol II was used for comparison across all products tested, regardless of their base composition. Gynol II is a spermicide and thus is not a directly comparable benchmark but it is often included in studies investigating personal lubricants (grandfathered). - The benchmark was used to evaluate the toxicity of available products and together to assess the capacity of the *in vitro* system to be used for safety assessments. | Dezzutti et al., 2012 |
| Gel-based personal lubricants (12) | - N-9 aqueous solution (2%) - Universal Placebo (containing hydroxyethylcellulose, water, sodium chloride and sorbic acid) | HeLa cervical cells | - Cytotoxicity (LDH) - Osmolality assessment - pH and buffering capacity measurements | Clinical data for the Universal Placebo Gel | - The products tested were marketed; their safety evaluation was assumed to have been conducted given the access of the consumers to the products (commercially available). - The Universal Placebo gel is considered a safety standard in vaginal microbicide drug delivery based on clinical data, which qualifies it as a benchmark with negative control properties. The cell-based assays indicated that the Universal Placebo gel had low toxicity (as 1:5 dilution), whereas 3 of the products tested induced significant toxicity *in vitro* even at low concentrations (1:100). - N-9 was included as a vaginal-relevant toxicity standard at a 2% concentration which is commonly found in spermicidal products. - A pair of low and high toxicity benchmarks (Universal Placebo gel and N-9) was used in this study to evaluate the toxicity of the products and together investigated the test system’s capacity for safety assessments. | Cunha et al., 2014 |
| Vaginal lubricants of various osmolality values:   - Iso-osmolal (4) - Hyper-osmolal (5) - Hypo-osmolal (1-water) | N-9 (3%) | 3D organotypic vaginal-ectocervical tissue model (EpiVaginal™ - VEC from MatTek Corporation) | - Histology - TEER - Tissue viability (%) by MTT endpoint | NA | - The MTT assay could not discriminate between products of different osmolality values in terms of induced irritation. - The results indicated that products with osmolality values greater than 4 times that of human vaginal fluid (evaluated at 370±40 mOsm/kg) markedly reduced epithelial barrier properties and induced damage in tissue structure. Reduced barrier integrity as measured by TEER was also associated with products with high osmolality values. - No epithelial damage was observed for hypo- or iso-osmolal lubricants (osmolality < 400 mOsm/kg). - Gynol II is a spermicide and thus is a directly comparable benchmark to the products within the same class; however, it is often included in studies investigating personal lubricants (grandfathered) as a control for irritation. | Ayehunie et al., 2017 |
| Ingredients of vaginal care products:   - Emulsifier (2) - Moisturizer (1) - Rheology modifier (3) - Skin repair active (1) - Soothing active (1) | - Cleansing solution 1 (identified as Benchmark 2 in the manuscript) based on lactic acid, magnesium laureth sulfate, disodium laureth sulfosuccinate, cocamidopropyl betaine, sodium laureth sulfate - Cleansing solution 2 (identified as Benchmark 3 in the manuscript) based on PEG-80 hydrogenated glyceryl palmate, cocamidopropyl betaine, magnesium laureth sulfate, polysorbate-20, sodium cocoyl aminoacids, disodium laureth sulfosuccinate | Test system based on cultivated A431 cells derived from a vulva epidermoid carcinoma | Tissue viability (%) by MTT endpoint | NA | - The ingredients were formulated in demineralized water, paraffin oil or paraffin oil + water, and were applied onto the tissues surface without dilution. The benchmarks were diluted 1:10 in demineralized water. The use of the 3D tissue model as test system allowed for application of neat materials, which otherwise could not have been tested in cell-based assays due to solubility issues. - Even though an irritation prediction model is not established in the manuscript, the authors evaluated Benchmark 2 as very slightly to slightly irritant, while Benchmark 3 was considered to be very slightly irritant. All ingredients investigated were considered non-irritant. - The benchmarks were included in the evaluation as comparators to the ingredients selected as relevant to vaginal care products, and in order to evaluate the test system’s use for safety assessments. | Roso et al., 2021 |

3D, three-dimensional (referring usually to tissue models); ET_50_, Effective Time necessary to reduce the viability of tissues to 50% of the viability of the negative control-treated tissues; HIV, Human Immunodeficiency Virus; IL, Interleukin; LDH, Lactate Dehydrogenase; MTT, 3-(4,5-dimethylthiazol-2-yl)-2,5-diphenyltetrazolium bromide; N-9, Nonoxynol-9; NA, Not Applicable; OTC, Over the Counter; PAS, Periodic Acid Schiff’s; TEER, Trans-epithelial electrical resistance; TEM, Transmission Electron Microscopy; TNF, Tumor Necrosis Factor

Note: The references are presented in chronological order and alphabetically within the same year (where applicable).
