# Supplementary material for: Defining benchmarking in the context of safety assessment of personal care and cosmetic products using New Approach Methodologies
Source: NAM J. 2026 Jul 7;2:100111. doi: 10.1016/j.namjnl.2026.100111 (PMC13382591; doi:10.1016/j.namjnl.2026.100111)
Supplement: Supplementary file 5 [file mmc5.docx]

**Supplementary Table 5.** Benchmarks used for safety assessment of oral care products.

| **Type of products tested** | **Benchmark(s) tested** | **Testing methodology** | | **Existing paired data** | **Data Summary** | **Reference** |
| --- | --- | --- | --- | --- | --- | --- |
|  |  | **Test system** | **Endpoint** |  |  |  |
| Commercially available:   - Adult dentifrices (6) - Baby toothpaste (2) - Children toothpaste (2) - Mouthwashes (3) - Oral cleansers (2) | Any of the products tested might be considered as potential benchmarks for their respective product line | 3D human buccal mucosal tissue model (EpiOral™ from MatTek Corporation) | - Cytokine expression (IL-1α, IL-1β) - Tissue Viability (MTT) | NA | - The products tested were marketed; their safety evaluation was assumed to have been conducted given the access of the consumers to the products (commercially available). - The commercial products may be considered benchmarks based on the anticipated irritation level, in this case driven in part by the age-category and associated sensitivity of the oral tissues (baby *vs.* children *vs.* adult) and by main ingredients (surfactants, alcohol, etc.). - Any products considered benchmarks can be used to evaluate the toxicity of prototypes of other similar products (that could be manufactured by competitors) and together to evaluate the capacity of the *in vitro* system to be used for safety assessments. | Klausner et al., 2007 |
| Mouthwash ingredients and finished products:   - 21.6% ethanol - 26.9% ethanol - COOL MINT®LISTERINE®   LISTERINE® | Potential benchmarks (even though not specified as such in the manuscript):   - COOL MINT®LISTERINE®   LISTERINE® | 3D human buccal mucosal tissue model (EpiOral™ from MatTek Corporation) | - Caffeine permeability - Ethanol transport and metabolism experiments - Histology - Tissue viability (MTT) | NA | - Tissue viability was not affected by any of the treatments applied topically. - No significant caffeine permeability was induced by any of the treatments. - Ethanol and ethanol-containing mouthwashes at concentrations used for these experiments did not induce any adverse effects compared to the controls nor had any apparent effect on the permeability of the standard model chemical caffeine. The results are as anticipated at least for the products tested that were marketed; their safety evaluation was assumed to have been conducted given the access of the consumers to the products (commercially available). - Even though the authors did not discuss the concept or applicability of benchmarks in this manuscript, the two commercial mouthwash products may be given consideration for this purpose. They are complex formulations, with presumed existing safety and consumer surveillance data and were evaluated as non-irritating to the oral mucosa (as anticipated based on the exposure protocol used) using an *in vitro* test system. In this scenario, these products may be used to evaluate a test system if considered benchmarks, and if clinical or other type of existing data sets are available for paired data analysis. | Koschier et al., 2011 |
| Formulas containing whitening actives:   - Sodium bicarbonate (1) - H_2_O_2_ (4): concentrations of 2%; 6%; 9%; 11.4% | Any of the tested formulas containing H_2_O_2_ might be considered as potential benchmark oral care lines based on this ingredient | 3D SkinEthic™ human reconstructed oral epithelium (RHO) and human gingival epithelium (RGE) from L’Oréal Research and Innovation Center | - Cytokine expression (IL-1α) - Histology - Tissue Viability (MTT) | NA | - Both 3D tissue models indicate the same rank order of cytotoxic effects for the formulas tested with the specification that the oral model (without a cornified layer) was notably more sensitive to the irritants than the gingival model. - In general, the exposure times resulting in increases in IL-1α release appeared to occur when notable reductions in viability as measured by MTT reduction occurred. - These buccal models may allow for the *in vitro* evaluation of the irritancy potential of teeth whitening products as well as other oral care products and cosmetics prior to clinical testing. | Wurzburger et al., 2011 |
| Commercially available whitening dentifrices:   - REMBRANDT^®^ GENTLE WHITE (surfactant-free) - REMBRANDT^®^ INTENSE STAIN^®^(contained pyrophosphate and cocamidopropyl betaine) - CREST^®^ 3D WHITE (contained pyrophosphate and sodium lauryl sulfate) | Any of the products tested might be considered as potential benchmarks | 3D human buccal mucosal tissue model (EpiOral™ from MatTek Corporation) | - Cytokine expression (IL-1α, IL-1β) - Tissue Viability (MTT) | NA | - Whitening toothpastes contain surfactants or surfactant blends that are added to solubilize flavor oils and improve cleaning performance. However, surfactants such as sodium lauryl sulfate are known to cause irritation *in vivo*. - The products tested were marketed; their safety evaluation was assumed to have been conducted given the access of the consumers to the products (commercially available). - Both REMBRANDT^®^ dentifrices (GENTLE WHITE and INTENSE STAIN, respectively) had longer ET_50_ values compared to the CREST^®^ 3D White dentifrice, thus indicating their reduced irritation potential as assessed by exposure to the *in vitro* model. Both surfactant-containing dentifrices show increased irritation potential compared to the surfactant-free dentifrice, however the irritation potential is reduced for the dentifrice containing cocamidopropyl betaine as a surfactant instead of sodium lauryl sulfate, which is a known irritant. - The test system was considered capable to rank order the products in terms of their irritation potential as anticipated by the components of the formulation. - Any of the products tested can be used to evaluate the toxicity of available products within the same product line or that are formulated using a surfactant-base or dentifrice-base. | Martinez et al., 2014 |
| Ingredients of vaginal care products:   - Emulsifier (2) - Moisturizer (1) - Rheology modifier (3) - Skin repair active (1) - Solubilizer (1) | Benchmark 1 based on alcohol, sodium benzoate, sodium monofluorophosphate, benzoic acid | Test system based on cultivated human normal gingival cells | Tissue viability (%) by MTT endpoint | NA | - The benchmarks were diluted 1:10 in demineralized water. The use of the reconstructed tissue model as test system allowed for application of neat materials, which otherwise could not have been tested in cell-based assays due to solubility issues. - Even though an irritation prediction model is not established in the manuscript, the authors evaluated Benchmark 1 as non-irritant to the gingival tissue model. All ingredients were considered non-irritant, except for the skin repair active which was evaluated to be non-irritant to very slightly irritant; the skin repair active was a blend of 3 acids (asiaticoside acide, madecassic acid and asiatic acid), which might explain the result. - The benchmarks were included in the evaluation as comparators to the ingredients selected as relevant to oral care products, and in order to evaluate the test system’s use for safety assessments. | Roso et al., 2021 |
| Mouth rinses (7 bases and 17 flavors) | Mouth rinse with known irritation potential (clinically demonstrated) | 3D human buccal mucosal tissue model (EpiOral™ from MatTek Corporation) | - Cytokine expression (IL-1α, IL-1β) - Tissue Viability (MTT) | Clinical data (examiner blind, single center, randomized, controlled, parallel design, 28-day study) using oral mucosal desquamation as endpoint – for the benchmark and several other prototypes | - A benchmark relevant to the product line tested was used to determine the irritation potential relative to a clinically tested formula and allow comparative ranking for prototype formulas. Prototypes were analyzed by demonstration of non-inferiority which is defined as the prototype mean result being statistically significantly higher than half the benchmark formula mean, using a one-side test at the 5% significance level. - The experiments confirmed inter-lab reproducibility of the benchmark in two separate labs, across 17 experiments (7 conducted in one lab and the other 10 in the second lab). - The protocol and test system used allowed the evaluation of irritation potential of mouth rinse prototypes prior to clinical testing and human use. The goal was to evaluate the impact flavor changes might have in clinically tested formulas. The *in vitro* tissue viability results trended with clinical oral mucosal exfoliation endpoint. | Reed et al., 2023 |

3D, three-dimensional (referring usually to tissue models); IL, Interleukin; MTT, 3-(4,5-dimethylthiazol-2-yl)-2,5-diphenyltetrazolium bromide; NA, Not Applicable

Note: The references are presented in chronological order and alphabetically within the same year (where applicable).
